# THE IMPACT OF MEDICARE ADVANTAGE ON SKILLED NURSING FACILITY FINANCIAL PERFORMANCE, STAFFING, AND QUALITY

**DOI:** 10.1093/geroni/igz038.2016

**Published:** 2019-11-08

**Authors:** John R Bowblis, Sean Huang

**Affiliations:** 1 Department of Economics, Miami University, Oxford, Ohio, United States; 2 Georgetown University, Washington, District of Columbia, United States

## Abstract

Since the mid-2000s, skilled nursing facilities (SNFs) face an increasing percentage of post-acute care patients enrolled in Medicare Advantage (MA), yet our understanding the of how this affects SNFs is limited. Managed care may provide better coordination and continuous care that enhances SNF quality, but MA plans can also negotiate lower payments, providing SNFs with fewer financial resources to invest in staffing and quality. We use data from 2011-2015 from Medicare Beneficiary Summary File, Minimum Data Set, Certification and Survey Provider Enhanced Reporting, and Medicare Cost Reports to estimate linear fixed effect panel regressions with instrumental variables. We find that SNFs with greater MA share of post-acute care admissions have worse financial performance, lower nursing staff levels, and worse quality as measured by deficiency scores. Our finding favors the hypothesis that MA creates downward financial pressure and strong MA presence in local markets can potentially to spillover to non-MA residents.

